# Improving the usability of open health service delivery simulation models using Python and web apps

**DOI:** 10.3310/nihropenres.13467.1

**Published:** 2023-10-05

**Authors:** Thomas Monks, Alison Harper

**Affiliations:** 1 University of Exeter Medical School, University of Exeter, Exeter, England, UK; 2 NIHR Applied Research Collaboration South West Peninsula, University of Exeter, Exeter, England, UK

**Keywords:** Open Science, Discrete-Event Simulation, Health Services Research, Web Applications, Python, Model Reuse, Reproducibility

## Abstract

One aim of Open Science is to increase the accessibility of research. Within health services research that uses discrete-event simulation, Free and Open Source Software (FOSS), such as Python, offers a way for research teams to share their models with other researchers and NHS decision makers.

Although the code for healthcare discrete-event simulation models can be shared alongside publications, it may require specialist skills to use and run. This is a disincentive to researchers adopting Free and Open Source Software and open science practices. Building on work from other health data science disciplines, we propose that web apps offer a user-friendly interface for healthcare models that increase the accessibility of research to the NHS, and researchers from other disciplines. We focus on models coded in Python deployed as streamlit web apps.

To increase uptake of these methods, we provide an approach to structuring discrete-event simulation model code in Python so that models are web app ready. The method is general across discrete-event simulation Python packages, and we include code for both simpy and ciw implementations of a simple urgent care call centre model. We then provide a step-by-step tutorial for linking the model to a streamlit web app interface, to enable other health data science researchers to reproduce and implement our method.

## Plain language summary

Simulation models provide a quantitative way for researchers to make predictions about complex health services, for example to assess the effects of changes to patient care pathways. The most common approach used for health services research is discrete-event simulation. Historically, research has used software that must be purchased and has restrictive licensing. This can make it difficult for other researchers, and NHS staff such as managers and clinicians, to use the model to help with their planning and resourcing decisions.

One aim of Open Science is to increase the accessibility of research. Free and Open Source Software (FOSS) such as Python offers a way for research teams to share their models with other researchers and NHS decision makers. Although the code for simulation models can be shared alongside publications, it may require specialist skills to use and run. Building on work from other health disciplines, we propose that web apps offer a user-friendly interface for healthcare models that increase the accessibility of research. A web app runs in the browser of a computer and allows users to update model parameters, run different experiments, and explore the impact on the health service that is being studied. We focus on a package called streamlit.

To increase uptake of these methods, we provide an approach to structuring model code in Python to enable the model to be easily integrated into streamlit. The method does not depend on a specific discrete-event simulation package. To illustrate this, we developed simulations using two Python packages called simpy and ciw of a simple urgent care call centre. We then provide a step-by-step tutorial for linking the model to the streamlit web app interface. This enables other health data science researchers to reproduce our method for their own simulation models and improve the accessibility and usability of their work.

## Introduction

Health service delivery is complex and costly. Computer simulation can be used to provide decision-support for design or re-design of healthcare processes, accounting for the inherent uncertainty associated with service delivery. Models that represent key characteristics or behaviours of a system or process are developed. Experimenting with the model enables exploration of the potential impact of changes to the system without the costs and risk associated with realworld changes ^1, 2^. In healthcare, discrete-event simulation (DES) is the most common simulation method for modelling ^3^. Reviews of the field demonstrate wide-ranging applications in health service delivery, for example evaluating operational performance, improving patient flow, and scheduling services ^4– 6^. Healthcare DES models are complex research artifacts: they are time-consuming to build, require clinical time and expertise, depend on specialist software and logic, and may be difficult to describe accurately using words and diagrams alone ^7^.

DES for health service delivery modelling has traditionally been conducted using proprietary software such as Arena, Simul8 and Anylogic ^4, 5, 8^. One reason is that proprietary simulation software is powerful, flexible, and user-friendly. For DES, proprietary licensed software have high costs per year, in the thousands, and restrictive licensing that limits how it can be used. This licensing causes two major issues for Open Science. The first is transparency of model artifacts ^9^. If a researcher, or in some cases the NHS, is required to purchase or subscribe to the software, and does not have the means, then they cannot scrutinize how models really work: they cannot reuse the artifact for further research, and in extreme cases are prevented from using a model to support the redesign of NHS services. In some cases software vendors may provide a free-to-use ”personal learning edition” of the simulation software. Here licensing allows researchers to inspect model code for their own learning, but legally prohibits use for further research or decision making. To avoid wasting the substantial clinical, and methodological expertise used in building models, new methods to improve the Open Science, transparency and usability of DES models are therefore of critical importance.

Free and Open Source Software (FOSS) for simulation offers an alternative to proprietary software without the licensing restrictions that affect uptake, sharing, and reuse of model code ^10, 11^. In health service delivery research, computational analyses, and simulation studies can be conducted using FOSS tools built in the popular coding languages of Python, R, and Julia ^12– 17^. Use of a language such as Python has further benefits for research as models can now be integrated with the wider data science ecosystem for statistical modelling and machine learning. Contemporary approaches are available to publish the exact software and code artifacts used in a study. These provide credit to authors, and a way to cite outputs other than research articles, for example, open science archives ( *e.g.* Zenodo, or the Open Science Framework), model specific archives ( *e.g.* the Peer Models Network ^18^ and CoMSeS Net ^19^ and online computational environments such as Code Ocean.

One downside of code based FOSS models is that the switch from commercial software can be challenging. In recent years there has been an effort to reduce this entry barrier with new resources and textbooks aimed at the Operational Research community ^11, 20, 21^. One open challenge is that these models are script-based and do not have have a user-friendly interface. In health service delivery research this may limit the uptake of new research tools for care pathway planning by NHS decision makers. This challenge is faced across all health data science disciplines and methods are beginning to emerge to enable research to progress using FOSS. For example, in health economics and pharmacology, robust methods have been proposed to build user-friendly interfaces for models developed in R using Shiny, an open source framework for building web applications in R ^22, 23^. There are few examples of simulation studies for health services delivery that have developed free and open source DES models with a focus on use and reuse, using R or Python ^14, 24, 25^. Several well-maintained DES libraries are available in Python, including simpy ^26^, salabim ^27^, and ciw ^28^, while streamlit provides a relatively simple alternative to *Shiny* for building web apps in Python.

In this paper we focus on Open Science for health service delivery research and, in particular, Operational Research (OR) studies that use DES to model health care pathways and service delivery using Python. This study is aimed at researchers already familiar with developing healthcare DES models in Python. We detail approaches to enhance open science in healthcare DES by developing a method to organise Python DES models and provide a user friendly interface for model users using streamlit for shareable web apps.

### Aims

This paper focuses on healthcare DES models developed using Python. It builds on pioneering work from health economics ^22^, pharmacology ^23^, and OR ^11, 20^ to support the community in adopting practices in R and Python FOSS tools to increase transparency and use/reuse of computational research artifacts. To complement existing work integrating Shiny in simulation models, we provide a method to add a streamlit interface to a Python model. We illustrate this with an application in DES using Python frameworks simpy ^26^ and ciw ^28^. We also detail a simple method for structuring Python DES models to enable reuse, extension, and swapping of simulation packages. Before presenting our method we provide an overview of FOSS, Python for computer simulation, and streamlit.

## Methods

We developed a method for structuring DES models to enable reuse and linkage to a user-friendly streamlit web app. The method allows users of healthcare models to quickly create and execute one or more experiments with a DES simulation model deployed via a web app using Python. Our work is designed to improve dissemination and implementation of DES research in health services to support patient care.

FOSS and Python have gained significant popularity and use in the UK’s NHS in recent years. This transformation has been inspired via communities such as NHS-Python (and NHS-R) as well as NHS-England promoting the use of FOSS tools ^29^. The Goldacre Review of research using health data ^30^ has called for shared, reusable code for data analysis, that minimises inefficient duplication and enables research continuity. FOSS grants the rights for users to adapt and distribute copies however they choose. FOSS software within health data science is provided with an open license.

We chose Python as it is consistently ranked in the top 4 of Stack OverFlows’s most used programming languages (4th in 2022) and top 5 most loved programming languages by developers. Python is also synonymous with modern data science and is used extensively in research and development of computational methods and applications. We selected streamlit, over alternatives, for example, dash or shiny for Python, due to relatively shallow learning curves, and speed at which new users can build and deploy models. Given the code-based nature of our work we illustrate the implementation of streamlit in the form of a step-by-step tutorial.

We describe the steps involved in setting up a Python simulation model so that it is ready to incorporate into a web app, allowing user interactive simulation, basic tabular results presentation, basic interactive chart creation to display results, and use of our methods to create a large number of simulation experiments and run them in a single batch.

Our method is designed to be general to any DES package in Python. We primarily illustrate our method using a model built in simpy. We illustrate how our method supports interchangeability of models using a second simulation package called ciw and provide a full implementation in our *Extended data* ^31^.

Although our case study model is simple, the methods we describe are applicable across more complex simulations. To illustrate, our online *Extended data* ^31^ include an application to a second case study with time dependent arrivals, and multiple activities and classes of patient.

### Case study: an urgent care call centre model

**Figure 1.**
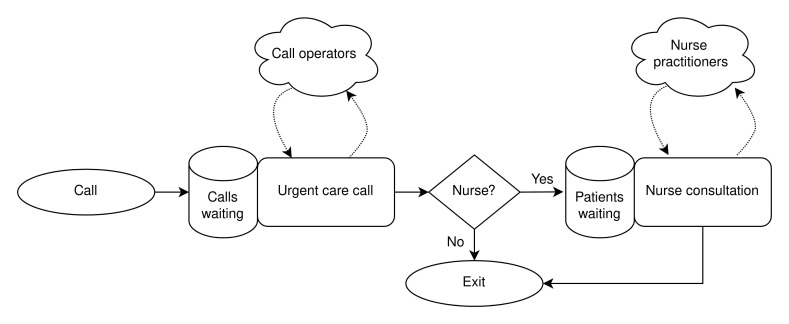
Urgent care call centre queuing model.

Our model is a stylised model of a typical urgent care call centre. Patient calls arrive at random to a call centre. Operators answer the calls, with a first in first out queue, triage the patient, interview following a standard script, and provide a call designation; for example, if the patient should travel to an emergency department, book an appointment in primary care with a General Practitioner (family doctor), or if a call back from a nurse is needed. If a caller needs to speak to a nurse they enter a first in first out queue until a nurse is available. Figure 1 illustrates patient flow in the model and use of resources.

### Simulation software: simpy

Simpy is a DES package implemented in Python. It uses a process-based simulation worldview. DES models are built by defining Python generator functions and logic to request and return resources. Although simpy provides a full DES engine, the package does not contain many of the additional features offered by a full commercial simulation package, such as Arena or Simul8, including a user interface, instead simpy offers a lightweight, flexible tool that can be integrated with the rest of the Python data science ecosystem. It has been used to model a wide variety of health condition pathways and healthcare operations; for example, the coronavirus disease 2019 (COVID-19) ^14, 32^, renal ^12^, stroke ^33, 34^, heart failure ^35^, cancer care ^36^, end of life care ^13^, and operating theatre management ^24, 37^.

### Web app technology: streamlit

Streamlit is a software package to build web applications (apps) in Python. It has been designed to make web apps accessible to non-experts. A typical streamlit application is a short Python script.

Web apps built with streamlit can be interactive. Deployment of models to users could be as simple as a local Python script that provides a browser based front-end to a model, hosting the app in streamlit’s free to use community cloud, or as complex as deployment to paid infrastructure such as Google Cloud. Our method is applicable across all of these deployment methods. For simplicity we detail the building of a simple browser-based front-end to a simulation model that is run on a laptop or desktop.

### Analysis environment

All code was written in Python 3.9.16. The DES model was implemented in simpy 4.0.1 and ciw 2.3.7. We used streamlit 1.23.0 for web apps. Manipulation and analysis of simulation results was conducted using *pandas* 2.0.2 ^38^ and *numpy* 1.25.0 ^39^. Charts were created using *matplotlib* 3.7.1 ^40^ and *plotly* 5.15.0.

The computational analyses were run on Intel i9-9900K CPU with 64GB RAM running Pop! OS 20.04 Linux.

### Reproducibility and availability of code

In addition to the code presented in this paper we provide a more detailed online tutorial in a Jupyter book ( https://health-data-science-or.github.io/simpy-streamlit-tutorial/ ). The code and book are archived using Zenodo ^31^. Users can also obtain code from out GitHub repository: https://github. com/health-data-science-OR/simpy-streamlit-tutorial .

### Overview of method

**Figure 2.**
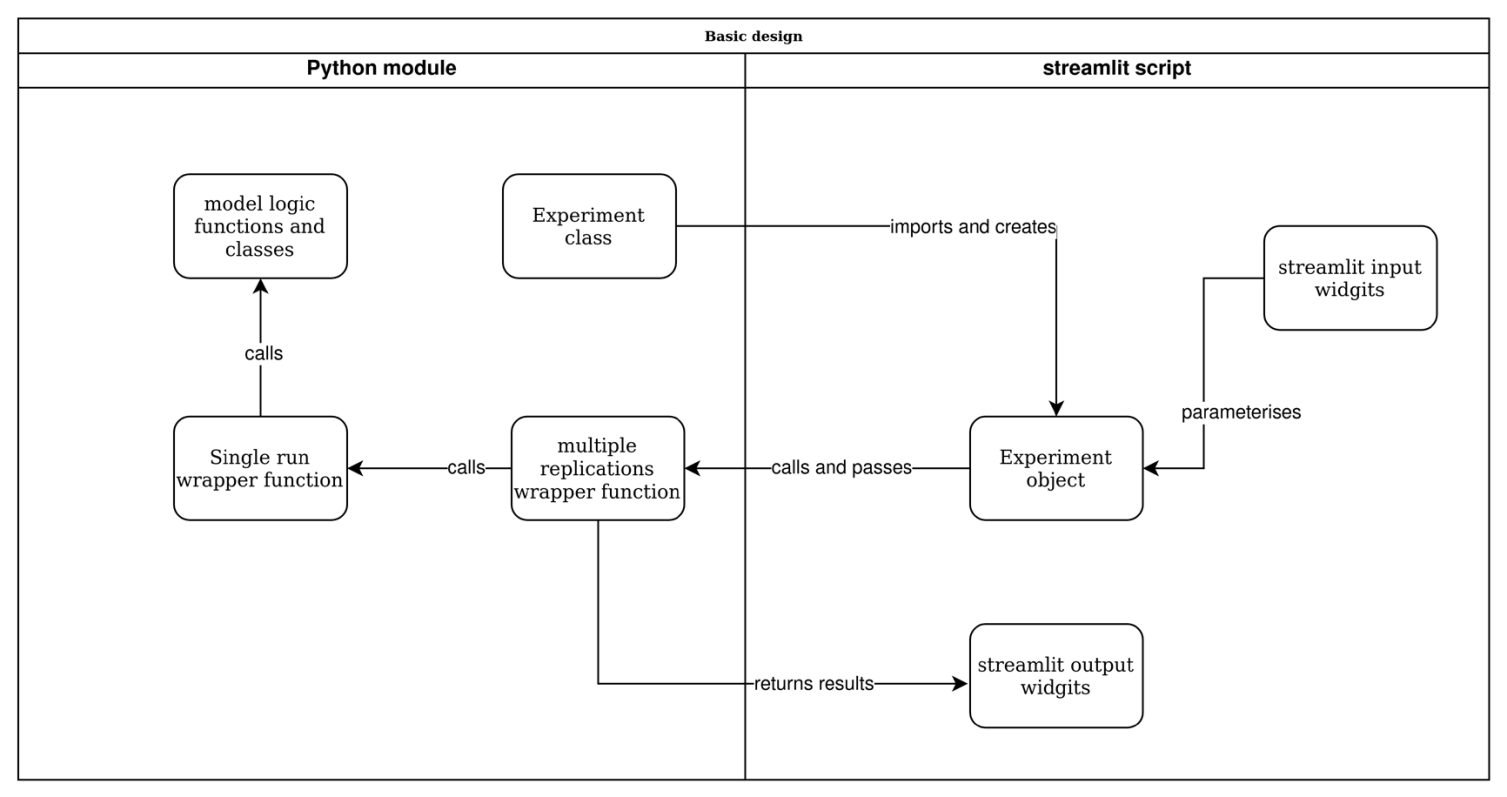
Model and web app structure overview.

As others have stated, a web application should allow a user to change parameter values and rerun the model ^22^. We add to this definition: a web app should provide a way for users to run more than one experiment at a time. By experiment we mean a change in one or more input parameters of the model. This type of experimentation is sometimes termed ’what-if’ analysis and is highly relevant to operational research in healthcare ^2^. Figure 2 illustrates the main concepts in the setup of our approach. We briefly outline these before describing implementation and code in our case study.

***Encapsulating model logic in a Python module***. Our method structures code and models so that a web app front-end does not depend on the simulation software package, or detailed logic of the model code. The opposite is also true, the implementation of the model is not affected by the logic or decisions made in the web app. Our approach ensures that a model is designed so that it can be run from a standard Python script, Jupyter notebook, or even a command line programme.

When combined with the model wrapper functions we propose in the next two sections, the simulation model becomes “swap-able”. This allows researchers to easily explore different implementations of a model in different software. For example, a model may have been developed in simpy, but is re-implemented in salabim or ciw without any change to the web app itself. To a lesser degree, this also simplifies the implementation of experiments that vary the process logic within a simulation as well as quantitative parameters; for example, modelling an emergency department with and without rapid assessment and treatment protocols as patients arrive. A caveat in this second case is that depending on the process logic chosen, the web app might require additional logic to vary the parameters that are available to users.

We propose two ways to encapsulate a full model in Python. The first is to create a standard Python module ( *e.g.* called model.py) that contains the simulation model and associated functions. *I.e.* model logic, functions, classes, and constants (for example, functions to generate patient arrivals to a health care service). Second, a Python package could be created and possibly deployed via the Python Package Index. Once installed the package acts very much like a module with a user importing functionality to run model experiments. Of these two methods the simplest to maintain in a modelling study is a Python module.

***Experiment class.*** In our approach, code that uses the simulation model does not directly import model logic. Instead it relies on an Experiment class and a wrapper function.

The Experiment class is tightly coupled to the design of the simulation model. It contains a configuration of the simulation model that can be executed to obtain results. Another way to describe an experiment is that it is a collection of input parameters for the simulation model. For example, in a study of a cancer pathway a user might set up two experiments: one containing the default number of screening slots on a given day and one containing an increment. The results of the experiments (such as patient waiting time and resource utilisation) can then be compared.

***Wrapping up a run of the model with single run().*** This function acts as a wrapper to run a simulated experiment and process end of run results. A key aspect of the design is that single_run() accepts an instance of Experiment. The code then sets up the model following the userset parameters, executes a single run of the model and returns results. Listing 1 provides pseudo code for the design of this function. It is indicative of the general pattern you will follow; the exact details will vary depending on the simulation package in use.


1 def  single_run (experiment, run_length):
2       setup_results_collection (experiment)
3       model = create_simulation_model (experiment)
4       run_results = run_model (model, run_length)
5       summary_results = process_results (run_results)
6       return summary_results


**Listing 1.** Pseudo code for single run

***multiple replications() wrapper function.*** The most likely usage scenario is that a model is run using multiple independent replications. This is a simple function that accepts an Experiment object and the number of replications to run. It then calls the single_run() function the appropriate number of times to create the distribution of results.

***The streamlit script.*** The script contains all of the logic for the web app; for example, user settings, results tables and information about the simulation model.

The script imports the Experiment class, and the multiple_replications() wrapper function from the simulation model module. This is all that is needed to enable the interaction between the front-end and the model.

***streamlit input widgets.*** The software has a number of standard input widgets available. For example, sliders, and text boxes to allow users to vary numeric values representing model parameters, and buttons to allow users to run models.

***Experiment object.*** The script creates one or more instances of Experiment. The experiment object is parameterised by the script. For example, in an urgent care call centre two key parameters might be the number of call operators who handle incoming patient calls and nurses on duty and available for urgent call backs. The streamlit script might vary these inputs using the two values contained in streamlit input sliders.

***Calling multiple replications().*** The interface is setup so that a user clicks on a streamlit button an d passes a parameterised instance of Experiment to multiple_replications() along with the number of replications to run.

***streamlit output widgets.*** The multiple_replications() returns some form of results to the streamlit script; for example, a pandas.Dataframe containing results of each replication. The streamlit script then uses a output widget *e.g.* a streamlit.dataframe to display the results to the user.

## Case study: model implementation

### Getting simpy and streamlit

The code examples in this study have been created using a conda virtual environment. An analogy for a virtual environment is a box within an operating system. Inside the box, a specific version of Python is installed along with specific versions of data science and simulation libraries such as simpy. There can be multiple virtual environments within one machine. The approach attempts to minimise software dependency conflicts between projects. Full details of how to install our environment, along with a supporting video, is available in our *Extended data* ^31^. We note if users are familiar with other virtual environment tools such as poetry then this can also be used.

### Simulation model logic

To recreate the model from scratch, see our *Extended data* ^31^ for step by step instructions. Here we provide an overview of the main functions used to implement model logic.

The function arrivals_generator() is a simpy process for creating patient arrivals to the urgent care call centre. It contains a loop that continually samples the time until the next arrival using the Exponential distribution. Each patient arrival is allocated a new service process.

The function service() defines all of the logic after a caller has arrived. This includes requesting and queuing for a call operator resource, sampling call duration from a Triangular distribution, sampling if a nurse call back is required from a Bernoulli distribution, requesting and queuing for a nurse resource, and finally sampling nurse call duration from Uniform distribution. The function service() also collects patient waiting time and resource utilisation as the simulation executes.

### Coding the Experiment class

The Experiment class encapsulates a set of input parameters that configure the simulation model. The overall design of a class representing an experiment will depend on the simulation study, and to some extent user preference. Here we advocate one key design principal:

- Make use of default values for input parameters, either from constant variables, or read in from file.

Lines 5–15 of Listing 2 illustrate the use of default parameters when creating an instance of Experiment; for example, N_NURSES provides a default number of nurses. In the implementation these are variables with module level scope. They are combined with Python’s optional arguments for methods. This approach means that it is simple to create experiments with default or new parameter values. Listing 3 illustrates the method across three experiments: an experiment using all defaults; an experiment that varies the number of call operators; and an experiment that varies the number of call operators and nurses.


 1 class  Experiment:
 2      ’’’
 3      Parameter class for simulation model
 4      ’’’
 5      def __init__(self, n_operators=N_OPERATORS,
 6                     n_nurses=N_NURSES,
 7                     mean_iat=MEAN_IAT,
 8                     call_low=CALL_LOW,
 9                     call_mode=CALL_MODE,
10                     call_high=CALL_HIGH,
11                     chance_callback=CHANCE_CALLBACK,
12                     nurse_call_low=NURSE_CALL_LOW,
13                     nurse_call_high=NURSE_CALL_HIGH,
14                     arrival_seed=None, call_seed=None,
15                     callback_seed=None, nurse_seed=None):
16          ’’’
17          The init method sets up our defaults, resource
18          counts, dists, + result collection objects.
19          ’’’
20          # no. resources
21          self.n_operators = n_operators
22          self.n_nurses = n_nurses
23
24          # create distribution objects
25          self.arrival_dist = Exponential (mean_iat,
26                                                arrival_seed)
27          self.call_dist = Triangular (call_low, call_mode,
28                                           call_high, call_seed)
29
30          self.callback_dist = Bernoulli (chance_callback,
31                                               callback_seed)
32
33          self.nurse_dist = Uniform (nurse_call_low,
34                                         nurse_call_high,
35                                         nurse_seed)
36
37          # resources
38          # these variable are placeholders.
39          self.operators = None
40          self.nurses = None
41
42          # initialise results to zero
43          self.init_results_variables()
44
45     def init_results_variables (self):
46          ’’’
47          Initialise all of the experiment variables used
48          in results collection. This method is called at
49          the start of each replication.
50          ’’’
51          # variable used to store results of experiment
52          self.results = {}
53          self.results[’waiting_times’] = []
54
55          # total operator usage time
56          self.results[’total_call_duration’] = 0.0
57
58          # nurse sub process results collection
59          self.results[’nurse_waiting_times’] = []
60          self.results[’total_nurse_call_duration’] = 0.0


**Listing 2.** Experiment class


1 default_experiment = Experiment()
2 extra_operator = Experiment(n_operators=14)
3 extra_operator_and_nurse = Experiment(n_operators=14, n_nurses=10)


**Listing 3.** Example usage of Experiment

### Coding the wrapper functions

The function single_run() is detailed in Listing 4. The function accepts an instance of Experiment and optionally a user specified simulation run length. Logically the function is a very simple wrapper for a single replication of the the model configured using the input parameters in the model. It returns a summary of the results from that replication ( *e.g.* the mean operator waiting time in the replication).


 1 def single_run (experiment,
 2                   rc_period=RESULTS_COLLECTION_PERIOD):
 3     ’’’
 4     Perform a single run of the model and return
 5     a dictionary of results
 6
 7     Parameters:
 8     -----------
 9     experiment: Experiment
10         The experiment/paramaters to use with model
11
12     rc_period: float, optional
13                 (default=RESULTS_COLLECTION_PERIOD)
14          Results collection period for simulation.
15
16     Returns:
17     --------
18     dict
19     ’’’
20     # results dictionary.Each KPI is a new entry.
21     run_results = {}
22
23     # reset all results variables to zero and empty
24     experiment.init_results_variables()
25
26     # environment is (re)created inside single run
27     env = simpy.Environment()
28
29     # create the resources
30     experiment.operators = simpy.Resource(env,
31                                                 experiment.n_operators)
32
33     experiment.nurses = simpy.Resource(env,
34                                             experiment.n_nurses)
35
36     # setup arrivals_generators as simpy process
37     env.process(arrivals_generator(env, experiment))
38     env.run(until=rc_period)
39
40     # end of run results: calculate mean waiting time
41     run_results[’01_mean_waiting_time’] = \
42          np.mean(experiment.results[’waiting_times’])
43
44     # end of run results: calculate mean operator utilisation
45     run_results[’02_operator_util’] = \
46         (experiment.results[’total_call_duration’] \
47          / (rc_period * experiment.n_operators)) * 100.0
48
49     # end of run results: nurse waiting time
50     run_results[’03_mean_nurse_waiting_time’] = \
51          np.mean(experiment.results[’nurse_waiting_times’])
52
53     # end of run results: calculate mean nurse utilisation
54     run_results[’04_nurse_util’] = \
55         (experiment.results[’total_nurse_call_duration’] \
56          / (rc_period * experiment.n_nurses)) * 100.0
57
58     # return the results from the run of the model
59     return run_results


**Listing 4.** single run() wrapper

In our simpy example, lines 27 – 38 creates a simulation environment, operator and nurse resources, sets up arrivals_generator() as a simpy process, and executes the model. Lines 45–56 are executed when the simulation is complete and calculate summary variables. Note these might preferably be refactored into an end_of_run_results() function for instances with a large number of model metrics.

The multiple_replications() function is simpler than single_run(). It is detailed in Listing 5. In line 25 a Python list comprehension is used to repeatedly loop and execute single_run() and create a Python list. Each item in the list is a dict containing the results of a single replication of the model. Lines 28 to 30 then format the results as pandas.Dataframe.


 1 def multiple_replications (experiment,
 2                                rc_period=RESULTS_COLLECTION_PERIOD,
 3                                n_reps=5):
 4      ’’’
 5      Perform multiple replications of the model.
 6
 7      Params:
 8      ------
 9      experiment: Experiment
10          The experiment/paramaters to use with model
11
12     rc_period: float, optional (default=
      DEFAULT_RESULTS_COLLECTION_PERIOD)
13          results collection period.
14          the number of minutes to run the model to collect results
15
16     n_reps: int, optional (default=5)
17         Number of independent replications to run.
18
19     Returns:
20     --------
21     pandas.DataFrame
22     ’’’
23
24     # loop over single run to generate results dicts in a python list.
25     results = [single_run (experiment, rc_period) for rep in range (n_reps)]
26
27     # format and return results in a dataframe
28     df_results = pd.DataFrame (results)
29     df_results.index = np.arange (1, len (df_results) +1)
30     df_results.index.name = ’rep’
31     return df_results



**Listing 5.** multiple replications() wrapper

### Code to test the model

Before moving onto building a web app it is recommended that a basic Python script is created to test the code. Listing 6 assumes that the model code and wrapper functions have been stored in a Python module called model.py. Lines 1 and 2 illustrate that all that a model user needs do is import Experiment and multiple_replications(). In our example, the user is only interested in varying the number of operators, nurses, and the chance of a callback. Line 15–20 then parameterise the experiment and execute the model.


 1 from model import (Experiment,
 2                      multiple_replications)
 3
 4 # set number of resources
 5 n_operators = 13
 6 n_nurses = 9
 7
 8 # set chance of nurse
 9 chance_callback = 0.4
10
11 # set number of replications
12 n_reps = 5
13
14 # create experiment
15 exp = Experiment(n_operators=n_operators,
16                     n_nurses=n_nurses,
17                     chance_callback=chance_callback)
18
19 # run multiple replications of experment
20 results = multiple_replications(exp, n_reps=n_reps)
21
22 # show results
23 print(results.describe())


**Listing 6.** A basic Python script

### Converting to an interactive web app

**Figure 3.**
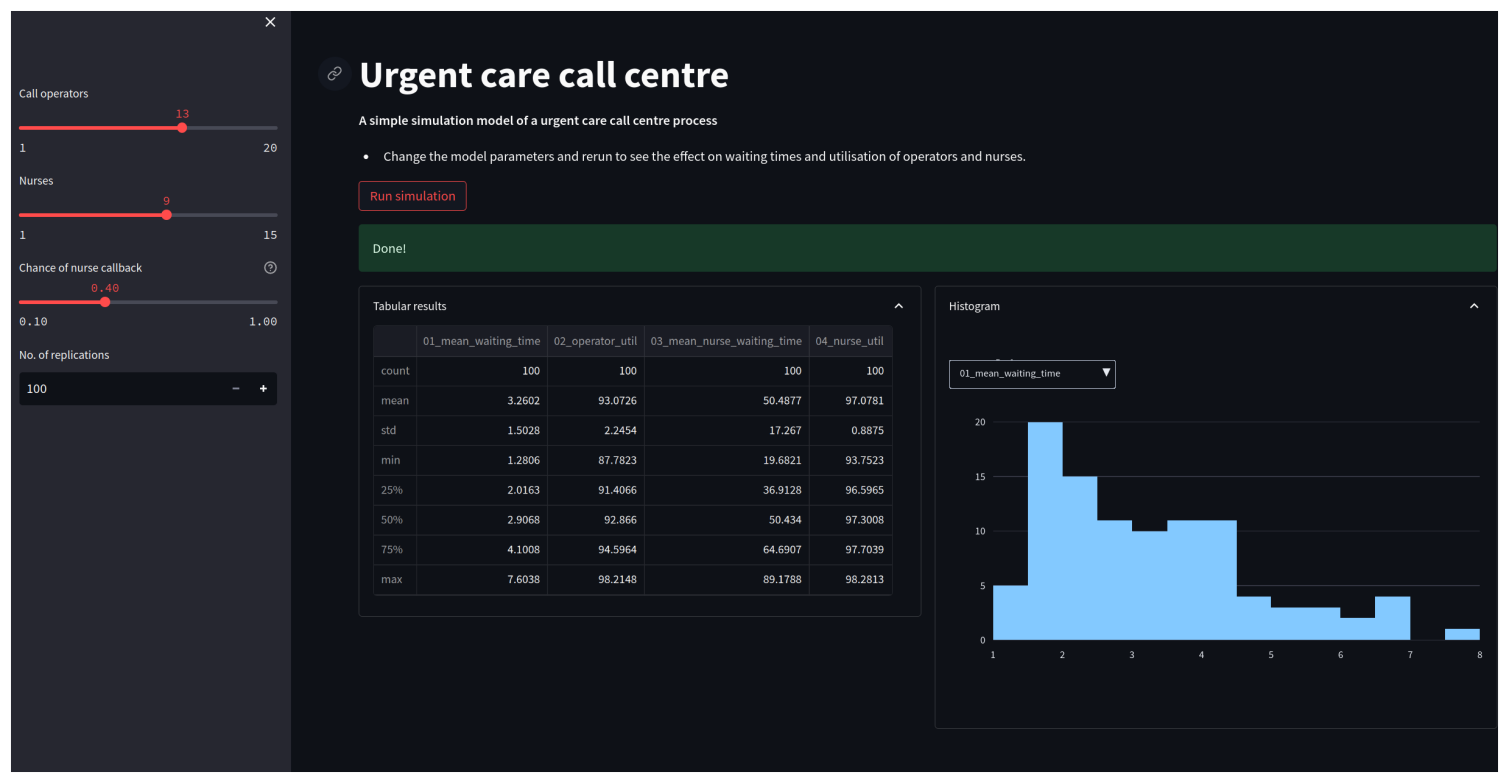
Urgent call centre DES web app. Shown is a screen shot of app running in a browser window. The app includes sliders for varying input parameters, and a interactive histogram to display results.

An example web app, for the urgent care call centre, is illustrated in Figure 3. In summary the web app provides the following functionality:

1. Sliders will allow a user to vary the number of operators, nurses, and the probability of a nurse callback;
2. For a given configuration, run an experiment of the model when a button is clicked;
3. The model will provide feedback to the user to let them know an experiment is running and when it has completed.
4. Display the summary statistics of the results of an experiment in a table and interactive histogram.

We also provide a title and some explanatory text for the model. For readers who wish to reproduce the web app, we recommend that Listing 6 is used as a starting point. Users can then incrementally makes changes to the script as we outline below. The script should be renamed basic_app.py.

### Step 1: import streamlit and create a title

At the top of the script add import streamlit as st. This will mean the script can now be launched as a streamlit app. We will also include a title for the app using st.title(‘Urgent care call centre’). The app can now be launched from the command prompt (terminal on Linux and Mac): streamlit run basic_app.py

### Step 2: Adding a button and displaying results

At the moment the model runs once - when the app starts. We will now add a st.button widget control when the app runs. In streamlit this is implemented using a Python if statement that checks the boolean value of the button. The use of a conditional may appear unintuitive at first, and is related to the way streamlit executes a script. We explain this in more detail in the next step.

After the model has executed the experiment, we need a way to display results to a user. We know that multiple_replications() returns a tabular result in the form of a dataframe. So we opt to use st.dataframe to display a formatted table to the user. This will appear underneath the button.

Listing 7 details the code from steps 1 and 2. The execution of the model is implemented in lines 28 to 34.


 1 # #################################################################
 2 # MODIFICATION: import streamlit
 3 import streamlit as st
 4 ###################################################################
 5 from model import Experiment, multiple_replications
 6
 7 # #################################################################
 8 # MODIFICATION: We add in a title for our web app’s page
 9 st.title ("Urgent care call centre")
10 ###################################################################
11
12 # set number of resources
13 n_operators = 13
14 n_nurses = 9
15
16 # set chance of nurse
17 chance_callback = 0.4
18
19 # set number of replications
20 n_reps = 5
21
22 # create experiment
23 exp = Experiment (n_operators=n_operators, n_nurses=n_nurses,
24                      chance_callback=chance_callback)
25
26 ###################################################################
27 # MODIFICATION: press a streamlit button to run the model
28 if st.button ("Run simulation"):
29
30      # run multiple replications of experment
31      results = multiple_replications (exp, n_reps=n_reps)
32
33      # show results
34      st.dataframe (results.describe ())
35 ###################################################################


**Listing 7.** basic app.py

### Step 3: Making the experiment interactive

We will now modify our app so that it is interactive; *i.e.*, a user will be able to vary the parameters on screen to run custom experiments. We will do this using streamlit.slider and st.number_input widgets. Both of these functions display a streamlit input widget and returns a numeric value that can be assigned to a variable. Listing 8 illustrates the creation of a slider and number input. The variables n_nurses and n_reps are the same data type as seen in Listing 7. The functions take several mandatory and optional parameters. For example with a slider we have provided the text to display to a user, the minimum and maximum values, the default value and the step size between one value and the next.


1 n_nurses = st.slider(’Nurses’, 1, 15, 9, step=1)
2 n_reps = st.number_input("No. of replications", 100, 1_000, step=1)


**Listing 8.** Input widgets

It is now essential to understand how streamlit executes a script. Each time a user updates the value of a slider or clicks the run button streamlit executes the full Python script file *i.e.* from top to bottom. This means, for example, that the integer value of n_nurses changes each time the slider is moved. We can now also make sense of the use of an if statement with the st.button function. When the button is clicked it is assigned the value True. streamlit then executes the full script and will also execute the conditional logic contained under the if statement. When a slider is changed the button has a value of False so the model will not run unnecessarily. Listing 9 details the code within the modified script.


 1 import streamlit as st
 2 from model import Experiment, multiple_replications
 3
 4 # We add in a title for our web app’s page
 5 st.title("Urgent care call centre")
 6
 7 # #################################################################
 8 # MODIFICATION: user experiment via app
 9
10 # set number of resources
11 n_operators = st.slider(’Call operators’, 1, 20, 13, step=1)
12 n_nurses = st.slider(’Nurses’, 1, 15, 9, step=1)
13
14 # set chance of nurse
15 chance_callback = st.slider(’Chance of nurse callback’, 0.1, 1.0,
        0.4,
16                                 step=0.05)
17
18 # set number of replications
19 n_reps = st.number_input("No. of replications", 100, 1_000, step=1)
20
21 # ##################################################################
22
23 # create experiment
24 exp = Experiment(n_operators=n_operators, n_nurses=n_nurses,
25                    chance_callback=chance_callback)
26
27 # A user must press a streamlit button to run the model
28 if st.button("Run simulation"):
29
30     # run multiple replications of experment
31     results = multiple_replications(exp, n_reps=n_reps )
32
33     # show results
34     st.dataframe(results .describe())


**Listing 9.** interactive app.py

### Step 4: Improving usability

To help usability, streamlit provides a number of simple features. We will implement three of these: a side bar that holds all of the input widgets, feedback to user that the model is running in the background, and a success box to report that the model run has completed.

A side bar is a section of the app that can be hidden or expanded depending on user preference, added using a Python with statement and st.sidebar. Its implementation can be seen in Listing 10 lines 10 to 21. Note that the widget code has been indented to ensure that it falls within the with statement block.

The second modification uses another with statement, this time combined with st.spinner. The spinner provides a simple animated prompt to users while the simulation is running. The code falls underneath the button logic for running the simulation model and can be seen in line 34 in Listing 10. Note again the indentation of the logic that will execute while the spinner is displayed on line 36. After the experiment is completed an st.success function is used to display a message of ‘Done’ to the user.


 1 import streamlit as st
 2 from model import Experiment, multiple_replications
 3
 4 # We add in a title for our web app’s page
 5 st.title ("Urgent care call centre")
 6
 7 # #################################################################
 8 # MODIFICATION: side bar
 9
10 with st.sidebar:
11
12      # set number of resources
13      n_operators = st.slider (’Call operators’, 1, 20, 13, step=1)
14      n_nurses = st.slider (’Nurses’, 1, 15, 9, step=1)
15
16      # set chance of nurse
17      chance_callback = st.slider (’Chance of nurse callback’, 0.1,
       1.0, 0.4,
18                                        step =0.05)
19
20      # set number of replications
21      n_reps = st.slider ("No. of replications", 5, 100, step=1)
22
23 # #################################################################
24
25 # create experiment
26 exp = Experiment (n_operators=n_operators, n_nurses=n_nurses,
27                      chance_callback = chance_callback)
28
29 # A user must press a streamlit button to run the model
30 if st.button ("Run simulation"):
31
32      # #############################################################
33      # MODIFICATION: add a spinner and then display success box
34      with st.spinner (’Simulating the urgent care system ...’):
35           # run multiple replications of experment
36           results = multiple_replications (exp , n_reps = n_reps)#
37
38      st.success (’Done!’)
39      # #############################################################
40
41      # show results
42      st.dataframe (results.describe ())


**Listing 10.** tidy app.py

### Step 5: Interactive visualisation of results

There are many options for visualisation in Python: *e.g.* matplotlib, plotly, altair, or bokeh. These are not specific to streamlit, and a full introduction to these packages is out of scope. Here we provide a simple example chart using plotly: a free and open source library for interactive charting. A matplotlib chart would be a fine choice as an alternative; however, we opt for an interactive chart to maximise the benefits offered from a web app. Our supplementary material details the coding of a function called create_user_filtered_hist. The chart is illustrated on the right hand side of Figure 3. The function accepts the results dataframe returns from the experiment. The histogram chart displays the distribution of a single performance measure across replications. The user is able to select a performance measure displayed from the full list using a drop down list. Note this is all achieved through plotly and avoids any complexity issues with the persistence of simulation results in the web app.

Listing 11 provides an extract of a streamlit script that makes use of the *plotly* function (see *Extended data* for the full listing). In the script we provide two further approaches to clean up the user interface and improve usability. We first use set_page_config to set the layout to ‘wide’ (lines x to y). We then create two columns for side by side result display using st.columns(2) (line x). We place the results table andplotly chart in these columns.


 1 if st.button("Run simulation"):
 2
 3      with st.spinner(’Simulating the urgent care system...’):
 4           results = multiple_replications(exp, n_reps=n_reps)
 5     st.success(’Done !’)
 6
 7     # create column layout
 8     col1, col2 = st.columns(2)
 9     with col1.expander(’Tabular results’, expanded=True):
10         # show tabular results
11         st.dataframe(results.describe())
12
13     with col2.expander(’Histogram’, expanded=True):
14         # create histogram and call plotly function
15         fig = create_user_filtered_hist(results)
16         st.plotly_chart(fig, use_container_width=True)


**Listing 11.** Modified results presentation code

## Additional functionality

### Loading and running multiple experiments

The Experiment class provides a simple way to run multiple experiments in a batch. To do so we can create multiple instances of Experiment, each with a different set of inputs for the model. These are then executed in a loop.

**Figure 4.**
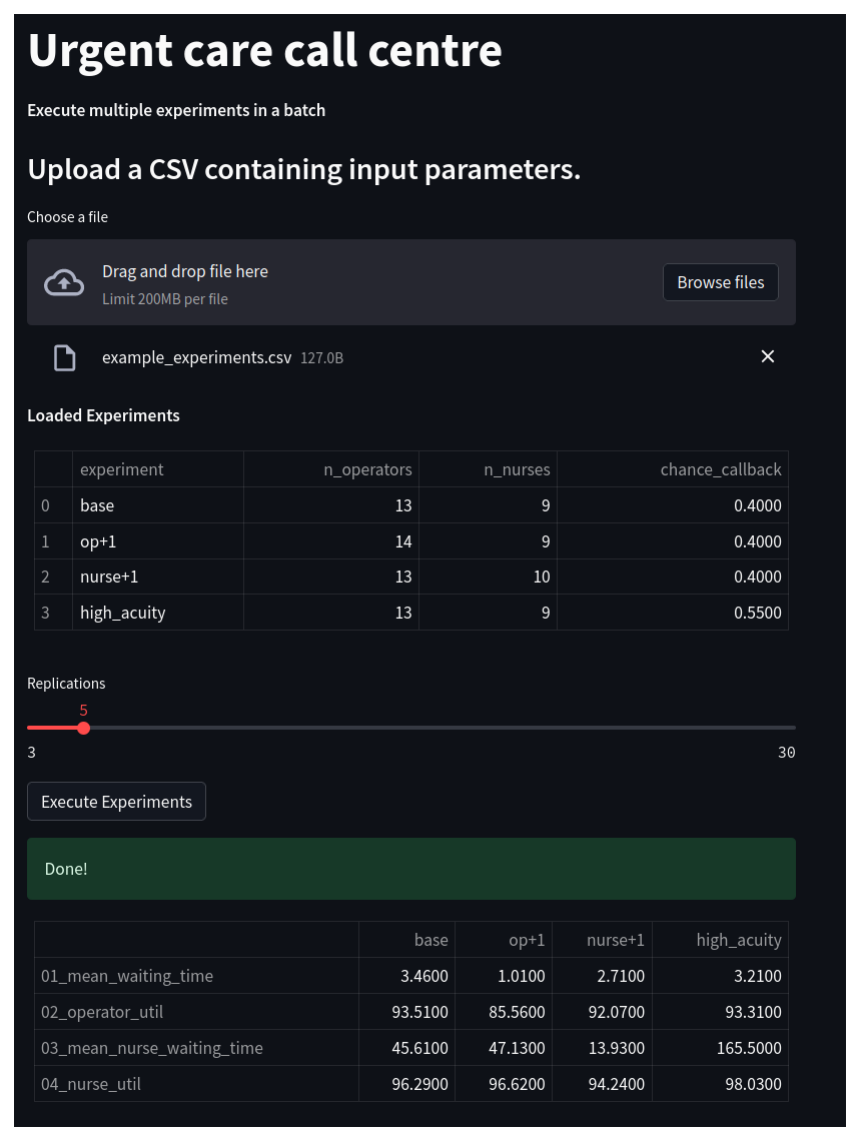
Multiple experiments app for the Urgent Care Call Centre. Shown is a screen shot of the app running in a browser window. The app has loaded and executed a CSV file containing four experiments. A table summarises experiment results.

A method to implement this in streamlit is to upload a Comma Separated Value (.CSV) file containing a list of experiments to the web app. This can be stored internally as a pandas.Dataframe and displayed using a streamlit widget such as st.table. A user can then edit experiment files locally on their own machine (for example, using a spreadsheet software, or using CSV viewer/editor extensions for Jupyter-Lab or Visual Studio Code) and upload, inspect, and run, and view results in the app. Example code to implement the approach is included in the *Extended data* ^31^. Figure 4 illustrates the web app it creates.

### Replacing simpy with ciw

Our DES model has been implemented in simpy, but our approach allows for this to be replaced if required. A high-quality alternative to simpy is ciw ^28^: a FOSS package for discrete-event simulation of queuing networks. A full overview of ciw is out of scope; we refer interested readers to its extensive online documentation.

To make the change we only need to update model.py. The streamlit script does not need to be modified. The main modifications are to the single_run() function - that now creates an instance of a ciw network model and runs it for one replication - and Experiment that now uses ciw’s built in distributions. See the the online *Extended data* ^31^ for full code listings. The ciw implementation provides qualitatively identical results and the same conclusions as simpy.

## Discussion

We demonstrate how to build a user-friendly interface to DES models coded in Python using streamlit. Our work aims to enhance Open Science within health service delivery and OR. Our approach supports researchers to share their computer models with other research teams and the NHS.

Strengths of our approach include a simple way to structure DES model Python code to allow for reuse, extension, and replacement, of models without the need to heavily modify a user interface. We demonstrated the latter by switching from simpy to ciw, although the approach would work just as well with different DES packages ( *e.g.* salabim). There may also be benefit in testing if the approach transfers to Agent Based Simulation.

The apps created in the previous sections can be shared with other researchers and the NHS using a very simple method: code and virtual environment files are committed to GitHub (or alternative such as GitLab). These can then be downloaded and run on a laptop or desktop. Python has an advantage that it is a cross-platform language, *i.e.* code will run on Microsoft Windows, Mac OS or Linux ^10^. Alternatively users can make use of streamlit community cloud, containerisation ( *.* using Docker); or creating an .exe file (on Microsoft Windows). There is also the more complex option to host the web app remotely and pay for more powerful computing infrastructure ( *e.g.* Google Cloud).

Another strength of our approach is that our multiple_replications function is easily parallelisable using Python’s *joblib* library. If the model is used locally, there may be a large time saving benefit of taking this approach, *i.e.* running parallel replications on a laptop with 20 virtual cores. However, in general these benefits do not transfer to web based models ^10^, *i.e.*, *streamlit’s* community cloud deployment will limit model execution to a single CPU.

When compared to commercial DES software packages that are commonly used in health research, such as Simul8, or AnyLogic, a limitation of our approach is that we do not display a dynamic patient pathway or queuing network that updates as the model runs a single replication. This is termed Visual Interactive Simulation (VIS) and can help users understand where process problems and delays occur in a patient pathway ^41^; albeit with the caveat that single replications can be outliers. A potential FOSS solution compatible with a browser-based app could use a Python package that can represent a queuing network, such as NetworkX ^42^, and displaying results via matplotlib. If sophisticated VIS is essential for a FOSS model then researchers may need to look outside of web apps; for example, salabim provides a powerful FOSS solution for custom animation of DES models.

## Conclusions

One aim of Open Science is to increase the accessibility of research. The code for healthcare DES models, often tackling important treatment pathways, can be shared alongside publications, but requires specialist skills to use and run. Building on work from other health data science disciplines, we propose that web apps offer a user-friendly interface for healthcare DES models that increases research accessibility to the NHS and researchers from other disciplines. We provide an approach to structuring DES model code in Python and a worked example linking this to a streamlit interface. Further work could consider interface design, and visual interactive simulation.

## Data Availability

A detailed online tutorial with executable code is available online ( https://health-data-science-or.github.io/simpy-streamlit-tutorial/). Source code available from: https://github.com/health-data-science-OR/simpy-streamlit-tutorial Archived source code at time of publication: https://doi.org/10.5281/zenodo.8193001 ^31^. License: MIT License

## References

[1] Robinson S Nance RE Paul RJ : Simulation model reuse: definitions, benefits and obstacles. *Simul Model Pract Theory.* 2004;12(7–8):479–94. 10.1016/j.simpat.2003.11.006

[2] Pitt M Monks T Crowe S : Systems modelling and simulation in health service design, delivery and decision making. *BMJ Qual Saf.* 2016;25(1):38–45. 10.1136/bmjqs-2015-004430 26115667

[3] Salleh S Thokala P Brennan A : Simulation Modelling in Healthcare: An Umbrella Review of Systematic Literature Reviews. *Pharmacoeconomics.* 2017;35(9):937–949. 10.1007/s40273-017-0523-3 28560492

[4] Vázquez-Serrano JI Peimbert-García RE Cárdenas-Barrón LE : Discrete-Event Simulation Modeling in Healthcare: A Comprehensive Review. *Int J Environ Res Public Health.* 2021;18(22): 12262. 10.3390/ijerph182212262 34832016 PMC8625660

[5] Forbus JJ Berleant D : Discrete-Event Simulation in Healthcare Settings: A Review. *Modelling.* 2022;3(4):417–33. 10.3390/modelling3040027

[6] Roy S Venkatesan SP Goh M : Healthcare services: A systematic review of patient-centric logistics issues using simulation. *J Oper Res Soc.* 2021;72(10):2342–64. 10.1080/01605682.2020.1790306

[7] Monks T Currie CSM Onggo BS : Strengthening the reporting of empirical simulation studies: Introducing the STRESS guidelines. *J Simulation.* 2019;13(1):55–67. 10.1080/17477778.2018.1442155

[8] Monks T Harper A : Computer model and code sharing practices in health-care discrete-event simulation: a systematic scoping review.OSF Preprints;2023. Reference Source

[9] Pouwels XGLV Sampson CJ Arnold RJG : Opportunities and Barriers to the Development and Use of Open Source Health Economic Models: A Survey. *Value Health.* 2022;25(4):473–479. 10.1016/j.jval.2021.10.001 35365297

[10] Byrne J Heavey C Byrne PJ : A review of Web-based simulation and supporting tools. *Simul Model Pract Theory.* 2010;18(3):253–76. 10.1016/j.simpat.2009.09.013

[11] Dagkakis G Heavey C : A review of open source discrete event simulation software for operations research. *J Simulation.* 2016;10(3):193–206. 10.1057/jos.2015.9

[12] Allen M Bhanji A Willemsen J : A simulation modelling toolkit for organising outpatient dialysis services during the COVID-19 pandemic. *PLoS One.* 2020;15(8): e0237628. 10.1371/journal.pone.0237628 32790773 PMC7425906

[13] Chalk D Robbins S Kandasamy R : Modelling palliative and end-of-life resource requirements during COVID-19: implications for quality care. *BMJ Open.* 2021;11(5): e043795. 10.1136/bmjopen-2020-043795 34035095 PMC8154294

[14] Anagnostou A Groen D Taylor SJE : FACS-CHARM: A Hybrid Agent-Based and Discrete-Event Simulation Approach for Covid-19 Management at Regional Level.In: *2022 Winter Simulation Conference (WSC).*IEEE;2022;1223–34. 10.1109/WSC57314.2022.10015462

[15] Wood RM Moss SJ Murch BJ : Optimising acute stroke pathways through flexible use of bed capacity: a computer modelling study. *BMC Health Serv Res.* 2022;22(1): 1068. 10.1186/s12913-022-08433-0 35987642 PMC9392305

[16] Mohd S Mustafee N Madan K : Leveraging Multi-tier Healthcare Facility Network Simulations for Capacity Planning in a Pandemic. 2021. 10.2139/ssrn.3794811 PMC10290165 38620120

[17] Crowe S Grieco L Monks T : Here’s something we prepared earlier: Development, use and reuse of a config- urable, inter-disciplinary approach for tackling overcrowding in NHS hospitals. *J Oper Res Soc.* 2023;1–16. 10.1080/01605682.2023.2199094

[18] Harvard S Adibi A Easterbrook A : Developing an Online Infrastructure to Enhance Model Ac- cessibility and Validation: The Peer Models Network. *Pharmacoeconomics.* 2022;40(10):1005–1009. 10.1007/s40273-022-01179-x 35907178

[19] Janssen MA Alessa LN Barton M : Towards a Community Framework for Agent-Based Modelling. *J Artif Soc Soc Simul.* 2008;11(2):6. Reference Source

[20] Knight V Palmer G : Applied Mathematics with Open-Source Software.Chapman & Hall/CRC Series in Operations Research;2022. 10.1201/9780429328534

[21] University of Exeter, Harper A Monks T : A Framework to Share Healthcare Simulations on the Web Using Free and Open Source Tools and Python.In: *Proceedings of SW21 The OR Society Simulation Workshop.*Operational Research Society,2023;250–60. Reference Source

[22] Smith R Schneider P : Making health economic models Shiny: A tutorial [version 2; peer review: 2 approved]. *Wellcome Open Res.* 2020;5:69. 10.12688/wellcomeopenres.15807.2 32904933 PMC7459889

[23] Wojciechowski J Hopkins AM Upton RN : Interactive pharmacometric applications using R and the shiny package. *CPT Pharmacometrics Syst Pharmacol.* 2015;4(3): e00021. 10.1002/psp4.21 26225240 PMC4394611

[24] Harper A Monks T Wilson R : Post-Covid Orthopaedic Elective Resource Planning using Simulation Modelling. *medRxiv.* 2023;2023–05. 10.1101/2023.05.31.23290774 PMC10748981 38135323

[25] Tyler JMB Murch BJ Vasilakis C : Improving uptake of simulation in healthcare: User-driven development of an open-source tool for modelling patient flow. *J Simul.* 2022;1–18. 10.1080/17477778.2022.2081521

[26] Team SimPy: SimPy 3.0.11.2020. Reference Source

[27] van der Ham R : salabim: discrete event simulation and animation in Python. *J Open Source Softw.* 2018;3(27): 767. 10.21105/joss.00767

[28] Palmer GI Knight VA Harper PR : An open-source discrete event simulation library. *J Simul.* 2019;13(1):68–82. 10.1080/17477778.2018.1473909

[29] NHS England: NHS England Open Source Programme. 2022. Reference Source

[30] Goldacre B Morely J : Better, broader, safer: using health data for research and analysis. A review commissioned by the Secretary of State for Health and Social Care. Department of Health and Social Care,2022. Reference Source

[31] Monks T Harper A : SimPy and StreamLit Tutorial Materials for Health-care Discrete-Event Simulation. *Zenodo.* [Data];2023. 10.5281/zenodo.8193001

[32] Bovim TR Gullhav AN Andersson H : Simulating emergency patient flow during the COVID-19 pandemic. *J Simul.* 2023;17(4):407–21. 10.1080/17477778.2021.2015259

[33] Ren Y Phan M Luong P : Application of a computational model in simulating an endovascular clot retrieval service system within regional Australia. *J Med Imaging Radiat Oncol.* 2021;65(7):850–7. 10.1111/1754-9485.13255 34105874

[34] Ren Y Phan M Luong P : Geographic Service Delivery for Endovascular Clot Retrieval: Using Discrete Event Simulation to Optimize Resources. *World Neurosurg.* 2020;141:e400–13. 10.1016/j.wneu.2020.05.168 32461178

[35] Wise AF Morgan LE Heib A : Modeling Of Waiting Lists For Chronic Heart Failure In The Wake Of The COVID-19 Pandemic.In: *2021 Winter Simulation Conference (WSC)*.2021;1–11. 10.1109/WSC52266.2021.9715505

[36] Richardson DB Cohn AEM : Modeling the Impact of Make-ahead Chemotherapy Drug Policies through Discrete-Event Simulation.In: *2018 Winter Simulation Conference (WSC)*.2018;2690–700. 10.1109/WSC.2018.8632236

[37] Hassanzadeh H Boyle J Khanna S : A discrete event simulation for improving operating theatre efficiency. *Int J Health Plann Manage.* 2023;38(2):360–79. 10.1002/hpm.3589 36271501

[38] McKinney W : pandas: a foundational Python library for data analysis and statistics. *Python for High Performance and Scientific Computing.* 2011;14. Reference Source

[39] van der Walt S Colbert SC Varoquaux G : The NumPy Array: A Structure for Efficient Numerical Computation. *Comput Sci Eng.* 2011;13(2):22–30. 10.1109/MCSE.2011.37

[40] Hunter JD : Matplotlib: A 2D graphics environment. *Comput Sci Eng.* 2007;9(3):90–5. 10.1109/MCSE.2007.55

[41] Bell PC O’Keefe RM : Visual interactive simulation: A methodological perspective. *Ann Oper Res.* 1994;53(1):321–42. 10.1007/BF02136833

[42] Hagberg AA Schult DA Swart PJ : Exploring Network Structure, Dynamics, and Function using NetworkX.In: Varoquaux G, Vaught T, Millman J, editors. *Proceedings of the 7th Python in Science Conference.*Pasadena, CA USA;2008;11–15. Reference Source

