# Supplementary material for: Improving the usability of open health service delivery simulation models using Python and web apps
Source: NIHR Open Res. 2023 Dec 15;3:48. Originally published 2023 Oct 5. [Version 2] doi: 10.3310/nihropenres.13467.2 (PMC10593330; doi:10.3310/nihropenres.13467.2)
Supplement: Peer review history

# Reviewer report: Reviewer response for version 2

**DOI:** 10.3310/nihropenres.14669.r30915

**Published:** 2023-12-28

**Authors:** Robert Smith

**Affiliations:** 1 The University of Sheffield, Sheffield, England, UK

The authors have addressed all of the points I raised. In particular I think having the app available is really useful to give people context. I hope that this paper is useful in pushing the OR community towards open-source programming languages (even if it is R rather than Python!). 

 I have a couple of other comments, which need no action:

 1) The authors stated that: "However, in general these benefits are unlikely to transfer to web based models ^10^ without using a paid tier offered by a web app hosting provider." However, this is not the case for R and shiny which I believe allows for analysis to be parallelized to multiple cores (although limited to a smaller number - I think maybe 4 or 8) on the free version of shinyapps.io.

 2) The authors state that "There is likely much that can be learnt from the Health Economic Evaluation community and research in R where models access secure data and parameters remotely using a secure API ^44^." This statement is true, but this functionality is not particularly commonplace, still being in early stages.

 3) "Further work could consider interface design, and visual interactive simulation." Couldn't agree more, there is a recognition in the health economics industry that more research on how to articulate models (in terms of UX design and visualizations) is needed.

 Thankyou again, I enjoyed reading this.

Is the rationale for developing the new method (or application) clearly explained?

Yes

Is the description of the method technically sound?

Yes

Are the conclusions about the method and its performance adequately supported by the findings presented in the article?

Partly

If any results are presented, are all the source data underlying the results available to ensure full reproducibility?

Yes

Are sufficient details provided to allow replication of the method development and its use by others?

Yes

Reviewer Expertise:

Application of methods from data-science to health economic evaluation.

I confirm that I have read this submission and believe that I have an appropriate level of expertise to confirm that it is of an acceptable scientific standard.

# Reviewer report: Reviewer response for version 2

**DOI:** 10.3310/nihropenres.14669.r30913

**Published:** 2023-12-28

**Authors:** Apostolos Kritikos

**Affiliations:** 1 Aristotle University of Thessaloniki, Thessaloniki, Greece

I want to congratulate the authors for their revision. I have no further comments.

Is the rationale for developing the new method (or application) clearly explained?

Yes

Is the description of the method technically sound?

Yes

Are the conclusions about the method and its performance adequately supported by the findings presented in the article?

Yes

If any results are presented, are all the source data underlying the results available to ensure full reproducibility?

Yes

Are sufficient details provided to allow replication of the method development and its use by others?

Yes

Reviewer Expertise:

I would like to clarify, for full transparency, that, coming from the Open Source Software Engineering discipline, my reviews focus on the proposed idea of using Open Source Software solutions to boost digital models, enable collaboration, and promote open science.

I confirm that I have read this submission and believe that I have an appropriate level of expertise to confirm that it is of an acceptable scientific standard.

# Reviewer report: Reviewer response for version 1

**DOI:** 10.3310/nihropenres.14611.r30520

**Published:** 2023-10-17

**Authors:** Apostolos Kritikos

**Affiliations:** 1 Aristotle University of Thessaloniki, Thessaloniki, Greece

The authors of this manuscript are conducting research on a very interesting challenge, that is, how to utilize open source software to provide an easy and user-friendly way for the health science community to work with models, share their findings and collaborate with other researchers or NHS decision makers.

 This is a well written manuscript and I very much enjoyed reading it and gained knowledge while doing so. I would like to clarify, for full transparency, that, coming from the Open Source Software Engineering discipline, my review will be focusing on the proposed idea of using open source software solutions to boost digital models, enable collaboration and promote open science.

**Pros**

- The manuscript is well written and structured. 
- The main point of the research is clear.
- The authors are successfully justifying their software choices and provide a detailed explanation on how their solution works and can be reproduced.
- The references are carefully selected and up to date.
- The assets (diagrams, code, examples) are easy to follow and made available as open data / open source code (i.e Zenodo, GitHub).

**Improvements**

- Reading the manuscript, the extensibility of the solution is evident when it comes to multiplicity of experiments and UI capabilities (within the limitations of the streamlit environment, of course). It is suggested that the authors clarify more on whether this solution can expand outside the DES models domain.
- Furthermore, could the proposed solution utilize another programming language (i.e. R). This is not being made very clear in the manuscript.

**Minor improvements**

- The "Plain Language summary" section is appearing twice in the manuscript. I apologize to the authors beforehand, if this is forced by the template of the publication.
- Discussion section also includes some limitations of the work. I would propose that the authors split "Discussions" section to "Discussion" and "Limitations or Threats to validity". I would like to further propose that the limitations section is extended a bit with other limitations (potentially linked with our suggestions on the Improvements section of this review - if deemed necessary by the authors).
- Finally, it is suggested that the last section, titled "Conclusions" should be renamed to "Conclusions and Future work" for more clear context.

Is the rationale for developing the new method (or application) clearly explained?

Yes

Is the description of the method technically sound?

Yes

Are the conclusions about the method and its performance adequately supported by the findings presented in the article?

Yes

If any results are presented, are all the source data underlying the results available to ensure full reproducibility?

Yes

Are sufficient details provided to allow replication of the method development and its use by others?

Yes

Reviewer Expertise:

I would like to clarify, for full transparency, that, coming from the Open Source Software Engineering discipline, my review will be focusing on the proposed idea of using open source software solutions to boost digital models, enable collaboration and promote open science.

I confirm that I have read this submission and believe that I have an appropriate level of expertise to confirm that it is of an acceptable scientific standard, however I have significant reservations, as outlined above.

# Response

**Published:** 2023-12-15

**Authors:** Thomas Monks

**Affiliations:** Exeter Medical School, University of Exeter, Exeter, England, UK

**Response to Reviewer 3 (R3)**

**R3: This is a well written manuscript and I very much enjoyed reading it and gained knowledge while doing so.**

*We are very grateful for a reviewer with your open source software experience to provide feedback on our work. Many thanks for your time.*

**R3: Reading the manuscript, the extensibility of the solution is evident when it comes to multiplicity of experiments and UI capabilities (within the limitations of the streamlit environment, of course). It is suggested that the authors clarify more on whether this solution can expand outside the DES models domain.**

*Yes, we do believe this is the case for health service delivery research where a variety of research methods are employed for modelling and prediction. Our primary experience with web apps is using computer simulation for modelling of health services, but we believe that it is equally application in other areas we have worked such as forecasting. That said, we think we should be cautious of making claims here without evidence (tests/applications). We thought a compromise was to focus on agent based simulation as has several characteristics similar to DES. We have added the following text in the section on strengths:*

- "Our method focus is DES, but the wrapper functions and experiment structure may generalise to other methods that use simulation. A closely related candidate method is Agent Based Simulation within healthcare as it makes use of stochastic sampling, multiple replications and experiments"

**R3: Furthermore, could the proposed solution utilize another programming language (i.e. R). This is not being made very clear in the manuscript.**

*Thank you for raising this important point. Yes we are confident the approach would generalise to R, although the technology and packages used would be different.  A study that greatly influenced our own is Smith and Schneider (2020) that is set in a Health Economic Evaluation and earlier in pharmacometrics Wojciechowski et al, (2015) contexts using Markov models. We have signposted readers to this work already (in the section on aims), but we have added the additional text in summary section of the discussion.*

- "Our Python method work is complementary to existing studies in Health Economic Evaluation and Markov modelling. Although no DES examples are available, modellers aiming to use an R DES package such as Simmer may wish to consult these sources for support (Smith and Schneider 2020; Wojciechowski et al, 2015)

**R3: The "Plain Language summary" section is appearing twice in the manuscript. I apologize to the authors beforehand, if this is forced by the template of the publication.**

*Thank you for identifying this problem with our manuscript. We think we made a mistake with the article template.  We believe this is now fixed and the PDF should only show one plain English summary.*

      

**R3: Discussion section also includes some limitations of the work. I would propose that the authors split "Discussions" section to "Discussion" and "Limitations or Threats to validity". I would like to further propose that the limitations section is extended a bit with other limitations (potentially linked with our suggestions on the Improvements section of this review - if deemed necessary by the authors).**

*We agree this improves the manuscript and will help readers understand the caveats of our work.  We have created a separate "limitations" section greatly expanded the discussion of limitations.  Please see our responses to other reviewers for details of these additions.*

**R3: Finally, it is suggested that the last section, titled "Conclusions" should be renamed to "Conclusions and Future work" for more clear context.**

*As requested we have updated the title of the final section to "Conclusions and Future Work"*

**References: **

- Smith R, Schneider P. Making health economic models Shiny: A tutorial.Wellcome Open Research. 2020;5(69)
- Wojciechowski J, Hopkins AM, Upton RN. Interactive pharmacometric applications using R and the shiny package. CPT: pharmacometrics & systems pharmacology. 2015;4(3):146-59.

# Reviewer report: Reviewer response for version 1

**DOI:** 10.3310/nihropenres.14611.r30528

**Published:** 2023-10-12

**Authors:** Robert Smith

**Affiliations:** 1 The University of Sheffield, Sheffield, England, UK

This is a very useful paper and the accompanying materials are very thorough. As someone who has advocated for web-based UIs for models in health economics its great to see the same methods applied in other areas (here OR). I hope this paper gets as much engagement and enthusiasm as we have benefitted from in health economics.

 I tend to code in R more than Python, so my comments are more high level.

1. It would be useful to have deployed the app so that those that are not familiar with these web-based applications can get an idea of the output from the code (we did this in Smith & Schneider, 2020 and I think it did help with engagement to have people play & share the app they'd built online). This is described in the discussion as "There is also the more complex option to host the web app remotely and pay for more powerful computing infrastructure (e.g. Google Cloud)" but this complexity is all on the developer side, and it makes it much easier for the user (who may not understand Python/GitHub and may not be able or permitted to install onto their machine). The paper also mentions 'Web-apps'  a large number of times  and then therefore its surprising there wasn't more discussion of how to achieve this.
2. Related to the above, there could be much more discussion about the best way to share the interactive model with potential users. Some options are listed, but there are trade-offs involved, especially in relation to data-sharing, something that we tried to solve in (Smith, Schneider & Mohammed., 2023) https://wellcomeopenresearch.org/articles/7-194 using APIs to send models to data, rather than data being sent to models. I don't think we've really cracked it, or explained it particularly well to those who are not familiar with such methods. Therefore we are still exploring different solutions, as this is probably the biggest barrier to modelling transparency in our industry (and I suspect it is the same in healthcare OR). I would be keen to hear the authors' opinions & experiences in Healthcare Operational Research settings and potential solutions.
3. There is no discussion around documentation and testing of code. While I appreciate this is not the primary focus of the paper, if the authors are advocating for sharing the code via GitHub and users deploying the apps themselves (e.g. locally in on organization servers) then model documentation & testing will be crucial (this is perhaps less crucial when the original authors deploy the app themselves  - although remains best practice). We have just written a paper on building R packages for health economic models (Smith, Mohammed & Schneider, 2023) https://wellcomeopenresearch.org/articles/8-419 with the aim being that each model should be documented and tested by the original authors in a systematic way before being shared with external parties, I assume there is a similar expectation in OR and with models developed in Python?
4. "streamlit’s community cloud deployment will limit model execution to a single CPU." presumably this is just an issue with streamlit rather than a fundamental problem with web-based models. For example models deployed on AWS/Google Cloud can be run on multiple CPUs.
5. There is not much discussion around the customizability of the UI. Again, I accept this is not the primary focus of the paper but my experience is that organizations will require the user-interface to look and feel a certain way (often to match their brand) and therefore the feasibility of this using Streamlit may limit its uptake.

Is the rationale for developing the new method (or application) clearly explained?

Yes

Is the description of the method technically sound?

Yes

Are the conclusions about the method and its performance adequately supported by the findings presented in the article?

Partly

If any results are presented, are all the source data underlying the results available to ensure full reproducibility?

Yes

Are sufficient details provided to allow replication of the method development and its use by others?

Yes

Reviewer Expertise:

Application of methods from data-science to health economic evaluation.

I confirm that I have read this submission and believe that I have an appropriate level of expertise to confirm that it is of an acceptable scientific standard, however I have significant reservations, as outlined above.

## References

[1] : Packaging cost-effectiveness models in R: A tutorial. *Wellcome Open Research* .2023;8: 10.12688/wellcomeopenres.19656.1 10.12688/wellcomeopenres.19656.1

[2] : Living HTA: Automating Health Technology Assessment with R. *Wellcome Open Research* .2022;7: 10.12688/wellcomeopenres.17933.1 10.12688/wellcomeopenres.17933.1 35600003

[3] : Making health economic models Shiny: A tutorial. *Wellcome Open Res* .2020;5: 10.12688/wellcomeopenres.15807.2 69 10.12688/wellcomeopenres.15807.2 32904933 PMC7459889

# Response

**Published:** 2023-12-15

**Authors:** Thomas Monks

**Affiliations:** Exeter Medical School, University of Exeter, Exeter, England, UK

**Response to Reviewer 2 (R2)**

**R2: This is a very useful paper and the accompanying materials are very thorough. As someone who has advocated for web-based UIs for models in health economics its great to see the same methods applied in other areas (here OR). I hope this paper gets as much engagement and enthusiasm as we have benefitted from in health economics**

*Your work was very influential on our own and we are very grateful to receive feedback from someone who has already worked in this area.  Many thanks for your time to review the manuscript and for links to your recent work.  Your work on R packages is very similar to our own current thinking and early work in Python.*

     

**R2: It would be useful to have deployed the app so that those that are not familiar with these web-based applications can get an idea of the output from the code (we did this in Smith & Schneider, 2020 and I think it did help with engagement to have people play & share the app they'd built online). This is described in the discussion as "There is also the more complex option to host the web app remotely and pay for more powerful computing infrastructure (e.g. Google Cloud)" but this complexity is all on the developer side, and it makes it much easier for the user (who may not understand Python/GitHub and may not be able or permitted to install onto their machine). The paper also mentions 'Web-apps'  a large number of times  and then therefore its surprising there wasn't more discussion of how to achieve this.**

*Yes we agree this is a good idea.  We have deployed the web app via streamlit community cloud.  It can be accessed here: https://simpy-app-tutorial.streamlit.app/.  We have added the link to opening paragraph of the 'Convert to an interactive web app' section of manuscript. Please note that streamlit cloud will put the app to sleep if it is not used for period of time.  If this is the case, there will be a short delay while the app 'wakes up'.*

     

**R2: Related to the above, there could be much more discussion about the best way to share the interactive model with potential users. Some options are listed, but there are trade-offs involved, ...**

*We are actively working in this area. We have attempted to include a short summary of issues in the manuscript for you, but there are a lot of things to consider here and it steps outside of the scope/core message of the tutorial.  We have added some discussion and citation of relevant works in the limitations section of the manuscript.*

*We thought the best approach to handle your points was to expand the limitations section of the manuscript.  We have also signposted readers to your existing material.  The following text has been added:*

- "Our tutorial has a number of limitations.  One issue we do not consider are projects where data, used to derive parameters or the parameters themselves used by the simulation model, may need to be kept private due to governance or ethical reasons.  In these instances hosting a model interface on a service such as streamlit community cloud is more challenging.  This is a contemporary problem for the UK NHS.  Initiatives such as OpenSafely have emerged to tackle reproducible pipelines and could support deriving model parameters (Williamson et al, 2020). There is likely much can be learnt from the Health Economic Evaluation community and research in R where models access secure data and parameters remotely using a secure API   (Smith et al, 2022)."

**R2: ... especially in relation to data-sharing, something that we tried to solve in (Smith, Schneider & Mohammed., 2023) https://wellcomeopenresearch.org/articles/7-194 using APIs to send models to data, rather than data being sent to models. I don't think we've really cracked it, or explained it particularly well to those who are not familiar with such methods. Therefore we are still exploring different solutions, as this is probably the biggest barrier to modelling transparency in our industry (and I suspect it is the same in healthcare OR). I would be keen to hear the authors' opinions & experiences in Healthcare Operational Research settings and potential solutions.**

*Regarding model sharing when data are sensitive or commercial in nature. We think your approach of consultant accessing secure data via an API is an important contribution and likely to be highly relevant in the UK with the new NHS Secure Data Environments that are shortly coming online. We are working in a complementary area, but with a slightly different approach (at a less advanced stage) where we are looking to build models that may rely on private data, but will be run by the NHS as opposed to a consultant.*

*In summary, we have been thinking about this in terms of containerisation plus continuous integration and deployment pipelines.  Apologies if you are already familiar with the terminology that we explain below.  In summary, a container is a way to wrap up a deployable version of a Linux OS, system tools, FOSS simulation software, a DES model, and an browser based interface. A container registry (e.g. DockerHub) is an online repository that hosts the container image. Using containerisation software (e.g. podman or docker) this can then be pulled to a local machine (e.g. a laptop or server within an NHS Trust) and parameterised using private data - it does not matter what OS the user has installed on their local machine.  We used continuous integration through a service like GitHub as it allows us to submit updates to code/models and automatically run a set of unit tests to check for bugs we may have introduced in the new changes.  If these tests are passed, we automatically build and push a new version of the docker image to the container registry.  We have a book chapter of the general approach accepted for publication (due out next year). We have a pre-review copy of the chapter available as a pre-print https://osf.io/qez45/. This also contains a worked example and a containerised model you can try (you would need to install docker). When compared to your API approach a limitation of our method is that a user must "pull" updates.  For the moment we adopted a "release" approach to continuous deployment so that we can talk to users without confusion e.g. model version 1.0.0 versus model 1.0.1 (if a patch was issued for a bug). But we believe (but have not tested) that we can automate the update of the container with the latest image via a simple start-up script. The benefit is that we can easily provide accessible models with interfaces similar to those in this article, that we know will run on any machine, with very minor updates to our workflow.*

*We would be happy to collaborate in this area.*

**R2: There is no discussion around documentation and testing of code. While I appreciate this is not the primary focus of the paper, if the authors are advocating for sharing the code via GitHub and users deploying the apps themselves (e.g. locally in on organization servers) then model documentation & testing will be crucial (this is perhaps less crucial when the original authors deploy the app themselves  - although remains best practice). We have just written a paper on building R packages for health economic models (Smith, Mohammed & Schneider, 2023) https://wellcomeopenresearch.org/articles/8-419 with the aim being that each model should be documented and tested by the original authors in a systematic way before being shared with external parties, I assume there is a similar expectation in OR and with models developed in Python?**

*Yes we would consider it to be very important. From our previous review work (Monks and Harper, 2023) we know that that published DES models in health rarely have any instructions to run them. Our additional discussion in the limitations section of the manuscript now covers this point.  *

- "While we have discussed creating model interfaces and wrapper functions for Python DES models, we have not been prescriptive about how these models are logically structured internally or what else should be included.  Others have argued that standardisation of code and its organisation will benefit transparency and reuse in Health Economic Evaluation (Alarid-Escudero et al, 2019; Smith et al., 2023). This includes naming conventions, unit testing, and project documentation. The inclusion of comprehensive documentation has been identified as a primary motivator of reuse of code (den Eynden, 2016)."

    

**R2: "streamlit’s community cloud deployment will limit model execution to a single CPU." presumably this is just an issue with streamlit rather than a fundamental problem with web-based models. For example models deployed on AWS/Google Cloud can be run on multiple CPUs.**

*Yes that is correct. We have modified the text in the strength's section of the manuscript to the following*

- "However, in general these benefits are unlikely to transfer to web based models without using a paid tier offered by a web app hosting provider. For example, streamlit's community cloud or ShinyApps.io free tier deployment will limit model execution to a single CPU"

    

**R2: There is not much discussion around the customizability of the UI. Again, I accept this is not the primary focus of the paper but my experience is that organizations will require the user-interface to look and feel a certain way (often to match their brand) and therefore the feasibility of this using Streamlit may limit its uptake.**

*This is a good point to raise and one that will be important to NHS organisations. The short answer is yes, streamlit app appearance is very customisable (particularly compared to commercial simulation software). At this time we have not expanded our tutorial material to cover this point. We thought the best way to handle this point was to list it as a limitation of our tutorial and note the potential in this area. We have included the following text:*

- "Our tutorial focused on the mechanics of building a usable streamlit interface for DES models, but we have not described detailed customisation options for interfaces.  These might, for instance, be important for models where NHS or research funder branding are important. Basic options provided by streamlit include the ability to display NHS or funder logos using the st.image() function. More flexible and detailed control of appearance can be accessed via streamlit's theming options, e.g., controlling font, and background colour of different sections or pages."

    

**References**

 1. Monks, T & Harper, A. (2023). Computer model and code sharing practices in healthcare discrete-event simulation: a systematic scoping review, Journal of Simulation, DOI: 10.1080/17477778.2023.2260772

 2, Harper, A., Monks, T., & Manzi, S. (2023, October 10). Deploying Healthcare Simulation Models Using Containerization and Continuous Integration. https://doi.org/10.31219/osf.io/qez45

# Reviewer report: Reviewer response for version 1

**DOI:** 10.3310/nihropenres.14611.r30522

**Published:** 2023-10-11

**Authors:** Geraint Palmer

**Affiliations:** 1 Cardiff University, Cardiff, Wales, UK

This article provides a tutorial on how to use streamlit to turn discrete event simulation scripts written in Python into user friendly apps. The reason for this is to help facilitate open simulation software by removing a key drawback to code-based simulations, namely the need to understand the code in order to understand and manipulate the software. In particular it describes how to structure simulation code to be able to integrate nicely with streamlit.

 This is a very good paper on an important topic, and highlights one tool that can be useful in facilitating open and accessible software. I learnt a lot here, and have only some minor comments on the manuscript.

- For the code listings I recommend following following the PEP8 style guide (https://peps.python.org/pep-0008/). An easy way to do this is to run the script through Black, a Python linter (https://black.readthedocs.io/en/stable/). This will make the listings much more readable and readily accessible to those familiar with Python.
- The "Methods" section on page 6 has a nice description of 'swap-able' code segments. Can you also add, that in the language of software development, this would be called 'modular' code.
- In Step 5 on page 16, a description of 'set_page_config' is given, but this does not appear in the listings. Similarly, line numbers of not given for 'st.columns(2)', only placeholders.
- On page 11 under "Code to test the model", it would be good to also mention that automated testing of code, such as unittesting, is standard practice in software development (https://software.ac.uk/resources/guides/testing-your-software) and can be useful here too for simulation verification.
- Listing 1 is pseudocode, but written and formatted as Python code, which may be confusing to readers following the tutorial. Can this be formatted differently?
- I am not sure the section on "Simulation Model Logic" on page 8 contributes to the understanding of the overall methodology and is needed. Those unfamiliar with Simpy might still not understand after reading this, and this is not a paper on Simpy.

These are minor comments and overall this is a very useful article.

Is the rationale for developing the new method (or application) clearly explained?

Yes

Is the description of the method technically sound?

Yes

Are the conclusions about the method and its performance adequately supported by the findings presented in the article?

Yes

If any results are presented, are all the source data underlying the results available to ensure full reproducibility?

Yes

Are sufficient details provided to allow replication of the method development and its use by others?

Yes

Reviewer Expertise:

Operational research, simulation, Python

I confirm that I have read this submission and believe that I have an appropriate level of expertise to confirm that it is of an acceptable scientific standard.

# Response

**Published:** 2023-12-15

**Authors:** Thomas Monks

**Affiliations:** Exeter Medical School, University of Exeter, Exeter, England, UK

**Response to Reviewer 1 (R1)**

**R1 This is a very good paper on an important topic, and highlights one tool that can be useful in facilitating open and accessible software. I learnt a lot here, and have only some minor comments on the manuscript.**

*Thank you for your review and feedback. We are very grateful to you for giving experience and time to improve the manuscript. We are also glad that you learnt something new in our material.*

**R1: For the code listings I recommend following following the PEP8 style guide (https://peps.python.org/pep-0008/). An easy way to do this is to run the script through Black, a Python linter (https://black.readthedocs.io/en/stable/). This will make the listings much more readable and readily accessible to those familiar with Python.**

*Thank you for your advise to use Black. This is an excellent piece of software. We confirm that we have done as requested.  Please note that there are instances where we have deviated slightly from Black's reformatting in order to better fit in with the journal's formatting requirements. For example, in Listing 5 the Black recommended that Line 26 was not split (it is only 70 characters in length). This is because the journal article cannot accommodate the full length of the line and wraps to a unlabelled line below.  Instead we introduced a manual split in the article.  We checked all changes from Black auto-formatting with Flake8 to make sure we did not introduce new PEP8 violations.  We also checked and updated references to listing line numbers in the main text where needed.*

**R1: The "Methods" section on page 6 has a nice description of 'swap-able' code segments. Can you also add, that in the language of software development, this would be called 'modular' code**

*As requested we have described the code as modular.*

**R1: In Step 5 on page 16, a description of 'set_page_config' is given, but this does not appear in the listings. Similarly, line numbers of not given for 'st.columns(2)', only placeholders.**

*Thank you for identifying this mistake.  We have removed the reference to `set_page_config` as we only intended to show an extract of the full listing here. We have also inserted the correct line number for `st.columns(2)`*

**R1: On page 11 under "Code to test the model", it would be good to also mention that automated testing of code, such as unittesting, is standard practice in software development (https://software.ac.uk/resources/guides/testing-your-software) and can be useful here too for simulation verification.**

*This is a very good point. To get readers started we have direct them to the Turing Way's section on code testing (that covers different testing strategies) and Onggo and Karatas (2016) that introduces test driven development and unit testing in the context of (agent based) simulation. We have added the following test to the manuscript:*

 "In a real simulation project it is recommended that more extensive code testing and verification of model logic is conducted. The full breadth of testing strategies available is out of scope for this tutorial. Interested readers are directed to the code testing section of The Turing Way and in an introduction to Test Driven Development and Unit Testing in the context of computer simulation".

References:

- The Turing Way Community. (2022). The Turing Way: A handbook for reproducible, ethical and collaborative research. Zenodo. doi: 10.5281/zenodo.3233853.
- Bhakti Stephan Onggo, Mumtaz Karatas (2016). Test-driven simulation modelling: A case study using agent-based maritime search-operation simulation, European Journal of Operational Research, Volume 254, Issue 2, https://doi.org/10.1016/j.ejor.2016.03.050.

**R1: Listing 1 is pseudocode, but written and formatted as Python code, which may be confusing to readers following the tutorial. Can this be formatted differently?**

*This is a very good point. We agree that Listing 1 can be improved. We have modified Listing 1 to use more standard psuedocode notation such as SUBROUTINE and left arrows for variable assignment.*

**R1: I am not sure the section on "Simulation Model Logic" on page 8 contributes to the understanding of the overall methodology and is needed. Those unfamiliar with Simpy might still not understand after reading this, and this is not a paper on Simpy**

*Thank you for raising this point. We have considered this point and decided that we would like to keep the section in the manuscript.  The reason is that we would like to be able to clearly sign-post the reader to our Extended Material (and external website) where we have additional tutorial material that can be used to recreate and understand the simulation model implementation. We agree that the papers focus is not simpy, but we would like to keep a clear audit trail for reproducibility.*
